# Fluid Resuscitation in Septic Patients

**DOI:** 10.7759/cureus.44317

**Published:** 2023-08-29

**Authors:** Shahid Qayyum, Kamran Shahid

**Affiliations:** 1 Nephrology, Diaverum Dialysis Center, Wadi Al Dawasir, SAU; 2 Internal Medicine/Family Medicine, California Institute of Behavioral Neurosciences & Psychology, Fairfield, USA

**Keywords:** severe sepsis, colloid solutions, crystalloid solutions, liberal vs restricted approach to fluid resuscitation in sepsis and septic shock, fluid resuscitation in sepsis

## Abstract

Sepsis is a life-threatening organ failure caused by a dysregulated response to infection. Fluid resuscitation and vasopressors are used to maintain systolic blood pressure and organ perfusion. Fluid resuscitation can be done with liberal or restricted fluids as well as colloids or crystalloid fluids. This review analyses the evidence for the use of liberal or restrictive fluids and colloids or crystalloids for the management of sepsis. A methodical search was conducted across PubMed, Cochrane Library, and ScienceDirect, and the Preferred Reporting Items for Systematic Reviews and Meta-Analyses (PRISMA) 2020 guidelines were followed for this study. Randomized controlled trials and retrospective observational studies were included in this study. Liberal and restrictive fluid strategies were found to be comparable in efficacy, but restrictive fluid regimens had the added benefit of a lower incidence of fluid overload. Balanced crystalloids were safer and more effective when compared to normal saline. Albumin replacement was found to be safe and showed efficacy in reducing mortality in patients with sepsis and septic shock.

## Introduction and background

Sepsis is caused by a host’s dysregulated response to infection and can lead to life-threatening organ failure. The severity of sepsis and the degree of organ failure are assessed using the sepsis-related organ failure assessment (SOFA). A simplified version of SOFA was designed to aid in the rapid diagnosis of sepsis called quick SOFA (qSOFA). While qSOFA is not used to define sepsis, it aids in the prompt recognition of patients at risk as it does not require laboratory testing. The qSOFA scale has three parts, hypotension (systolic pressure ≤100 mmHg), tachypnea (respiratory rate ≥22 breaths per minute), and mental status (Glasgow coma scale <15) [1]. Septic shock, a subset of sepsis, is associated with a 40% increase in in-hospital mortality. It can be identified by the use of a vasopressor in order to maintain a mean arterial pressure (MAP) at ≥ 65 mmHg and serum lactate level >2 mmol/L without hypovolemia [2]. Sepsis and septic shock are the leading cause of mortality in the intensive care unit (ICU) [3].

According to the Surviving Sepsis Campaign, patients with hypoperfusion caused by sepsis or septic shock should receive at least 30 mL/kg of intravenous (IV) fluids within the first three hours. There are no current recommendations for the use of liberal or restrictive fluids for patients who continue to show signs of hypoperfusion after the initial resuscitation [4]. While liberal IV fluids can increase cardiac pre-load and stroke volume, it can also lead to extravasation of fluid leading to edema affecting multiple organ systems. Additionally, IV fluid boluses can narrow the gradient between arterial and venous pressures thereby further decreasing perfusion [5].

Current recommendations suggest an early administration of either a balanced crystalloid solution (lactated Ringer’s or Plasma-Lyte A) or saline (0.9% sodium chloride) [4]. The high concentration of chloride in saline may lead to hyperchloremia, metabolic acidosis, and renal vasoconstriction [6]. Balanced crystalloids on the other hand have an electrolyte configuration similar to plasma and have been found to achieve better clinical outcomes with significantly lower incidence of acute kidney injury [7]. Albumin has oncotic, anti-inflammatory, and antioxidant properties and can alter nitric oxide metabolism. These properties are especially important in patients with sepsis, and it has been shown to reduce morbidity and improve organ function in critically ill patients [8].

Given the variability of the IV fluid regimens being currently used in clinical practice, we conducted a systematic analysis with the aim to compare restrictive fluids and liberal fluid use for initial resuscitation. In addition, we will also compare whether a balanced crystalloid solution or normal saline provides better clinical outcomes. Lastly, we will assess the role of the addition of albumin to crystalloids in clinical outcomes.

## Review

Method

A thorough literature search was conducted using the Preferred Reporting Items for Systematic Reviews and Meta-Analyses (PRISMA) criteria. Full-text publications, paid and free, indexed in PubMed, Cochrane Library, and ScienceDirect were searched from inception to 2023, using the keywords "Sepsis”, “Crystalloids”, "Colloids" and “Albumin”. Table 1 provides the Medical Subject Headings (MeSH) strategy used.

**Table 1:** MeSH strategy MeSH: Medical Subject Headings

Full Mesh
(("Sepsis"[Majr]) OR "Shock, Septic"[Majr]) AND ( "Crystalloid Solutions/adverse effects"[Majr] OR "Crystalloid Solutions/therapeutic use"[Majr] )
(("Sepsis"[Majr]) OR "Shock, Septic"[Majr]) AND ( "Albumins/adverse effects"[Majr] OR "Albumins/therapeutic use"[Majr] )

*Study Selection * 

We looked for studies that compared various fluid regimens available in patients with sepsis or septic shock. We excluded all studies that were not available in the English language as well as animal studies, gray literature, case reports, book chapters, editorials, and systematic reviews. Articles were first screened using the titles and abstracts only. Any duplicates were found and removed, and the relevant publications were chosen by inspecting the full text. Articles in English and those from 2014 to 2023 were included. Articles about other types of shocks such as cardiogenic and hypovolemic as well as other critical illnesses were excluded. 

Data Extraction and Analysis

We extracted data based on author, year, study design, sample size, gender distribution, median age, source of infection, intervention, and outcomes measured. Data were extracted and cross-checked by both authors and any disputes were solved. A narrative synthesis was performed on the extracted data.

Quality Appraisal

We used the Cochrane Risk of Bias assessment tools for clinical trials [9] and the modified Newcastle Ottawa quality assessment scale for the observational study included in this review.

Results

Search Results

**Figure 1:**
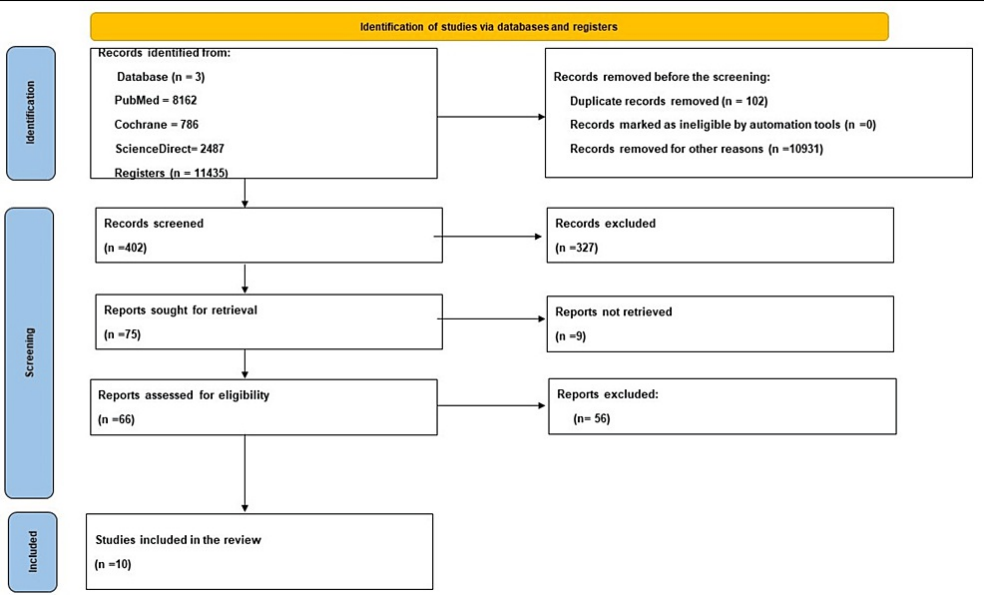
PRISMA flowchart of the literature and the search strategy PRISMA: Preferred Reporting Items for Systematic Reviews and Meta-Analyses

A thorough search of two databases yielded a total of 11435 articles. One hundred and two duplicates were removed and a further 10931 articles were excluded because they did not meet the inclusion criteria. Four hundred and two articles were screened using titles and abstracts of which 75 articles were sought for retrieval. Nine articles could not be retrieved so only 66 remaining articles were assessed for eligibility; only 10 were included in this review. Figure 1 shows the PRISMA flowchart of the literature and the search strategy of the studies [10].

Characteristics of the Included Studies

All included studies were published between 2014 and 2023. The following data were extracted from each article: author, year, study design, sample size, gender distribution, median age, source of infection, intervention, and outcomes measured. A summary of the characteristics of the included studies is shown in Table 2.

**Table 2:** Characteristics of included studies M: Male; F: Female; Pulm: Pulmonary; UTI: Urinary tract infection; GI: Gastrointestinal; IV: Intravenous; ICU: Intensive care unit; SBP: Systolic blood pressure; MAP: Mean arterial pressure; mmHg: Millimeter(s) of mercury; RRT: Renal replacement therapy; ED: Emergency department; SOFA: Sequential organ failure assessment

Author (Year)	Study design	Sample Size	Gender	Median age	Source of infection	Intervention	Measured outcome
Corl et al. (2019) [11]	Multi-center, randomized, unblinded, clinical trial	Total= 109 Restricted fluids= 55 Usual care= 54	M=50 F=59	Restrictive fluids= 71 Usual care= 73.5	Pulm= 30; UTI=29; GI=11; Skin/soft tissue=10	Restrictive group: 60ml/kg resuscitative IV fluids Usual care group= No specified limit	30- and 60-day all-cause mortality . Length of stay in ICU and hospital. New onset organ failure. Vasopressor-free days. Vasopressor and mechanical ventilation hours. Electrolyte abnormalities. Adverse events
Jessen et al. (2022) [12]	Multi-center, open-label randomized, parallel-group, unblinded, feasibility trial	Total=123 Restrictive fluids= 61 Standard care= 62	M= 71 F= 52	Restrictive fluids= 75 Standard care= 76	Pulm=88; UTI= 21; GI= 8; Skin/soft tissue= 4	Restrictive: Fluid bolus of 250ml isotonic crystalloids if hypoperfusion criteria were met, otherwise no fluids Standard care: Fluids by clinicians’ choice	Total volume of IV crystalloids administered in 24 hours. Feasibility measures. Serious adverse events and events within seven days. Total fluids (oral + IV) at 24 hours. Mechanical ventilation and vasopressor use within seven days. Acute kidney failure within seven days. 30- and 90-day mortality. Hospital length of stay.
Shapiro et al. (2023) [13]	Multi-center, randomized, unblinded superiority trial	Total= 1563 Restrictive fluid= 782 Liberal fluid= 781	M=826 F=737	Restrictive fluid= 59.1 Liberal fluid=59.9		Restrictive fluid: Fluid boluses up to 2L of total fluids if SBP < 100mmHg or MAP < 65mmHg ± 500ml rescue fluid Liberal fluid: 2l crystalloid infusion if SBP< 100mmHg or MAP <65mmHg ± rescue 500ml bolus	90-day all-cause mortality. Ventilator and vasopressor-free days. Days out of ICU and hospital. Initiation of mechanical ventilation. New-onset arrhythmias. Complications from catheter use. RRT free days.
Meyhoff et al. (2022) [14]	Multi-center, stratified, parallel-group, unblinded, open-label, randomized clinical trial	Total= 1531 Restrictive fluids= 755 Standard care= 776	M=904 F=627	Restrictive= 71 Standard care= 70	Pulm=415; UTI= 252; GI= 575; Skin/soft tissue= 126	Restrictive fluid= Fluids only given when the patient had severe hypoperfusion, to replace documented fluid losses, correct dehydration and electrolyte status, and ensure daily fluid intake of 1L Standard care= Fluids were given with no set upper limit and can be given as long as the patient had improvements in hemodynamics, to replace losses, and maintenance fluid according to the ICU protocol.	90-day all-cause mortality. One or more serious adverse effect. New episode of renal injury. Ventilator, life support, and RRT-free days.
Brown et al. (2019) [15]	Secondary analysis of the SMART trial [16]	Total= 1641 Crystalloids= 824 Saline= 817	M=899 F=742	Crystalloids= 60 Saline=60	Pulm= 353; UTI= 191; GI= 161; Skin/soft tissue= 71	Crystalloid: Before ICU, 1281 ±67 ml of crystalloid and 277±29ml of 0.9% saline. From ICU to discharge, 2967 ±4498 ml of crystalloids and 1374 ±3514 ml of 0.9% saline Saline: Before ICU, 1262 ±59 ml of 0.9% saline and 266± 32 ml of crystalloids. From ICU to discharge, 3454± 4982 ml of 0.9% saline and 629 ±2348 ml of crystalloids.	30- and 60-day in-hospital mortality. ICU, ventilator, vasopressor, and RRT-free days. Major adverse kidney events. Persistent renal dysfunction. New onset of RRT.
Jackson et al. (2020) [17]	Secondary analysis of the SMART trial [16]	Total= 1641 ICU-only: Total= 367 Crystalloids= 142 Saline=225 ED and ICU: Total= 1274 Crystalloids = 682 Saline= 592	M =899 F=742	ICU-only: Crystalloids= 58 Saline=60 ED and ICU: Crystalloids= 60 Saline= 59	Pulm= 353; UTI= 191; GI= 161; Skin/soft tissue= 71	ICU-only: Crystalloid group: 3570 ±5600ml of crystalloids and 1747 ±3088ml of saline in 30 days after ICU admission. Saline group: 918 ±2993ml of crystalloids and 3486 ±4534ml of saline in 30 days after ICU admission. ED and ICU group: Crystalloid group: 1109 ±1682 ml of crystalloids and 145 ±532 ml of saline before ICU admission. 2841± 4227 ml of crystalloid and 1297 ±3594 ml of saline after ICU admission. Saline group: 88 ±468 ml of crystalloids and 1135 ±1524 ml of saline before ICU admission. 520 ±2043 ml of crystalloids and 3442 ±5146 ml of saline after ICU admission	30-day in-hospital mortality. ICU, vasopressor, ventilator, and RRT-free days. Persistent renal dysfunction. Major adverse renal events. New onset of RRT.
Caironi et al. (2014) [18]	Multi-center, open-label, randomized, controlled trial	Total= 1810 Albumin= 903 Crystalloid= 907	M= 1093 F= 717	Albumin= 70 Crystalloids=69		Albumin: 300ml of 20% albumin till day 28 or ICU discharge and crystalloid solution when clinically indicated Crystalloid: Only crystalloid solution when clinically indicated	28- and 90-day all-cause mortality. Organ dysfunction and degree of dysfunction assessed using SOFA score. Length of ICU and hospital stay. RRT usage. Incidence of acute kidney injury. Ventilation days. Vasopressor-free days
Zhou et al. (2021) [19]	Retrospective observational study	Total= 7517 Crystalloids alone= 6597 Early combination= 920	M= 3866 F= 3651	Crystalloids= 67 Early combination= 67			28- and 60-day mortality. Length of ICU and hospital stay. Total volume of crystalloids given in ICU.
Geng et al. (2022) [20]	Meta-analysis	Severe sepsis= 5124 Septic shock= 3482				4-5% albumin= five studies 20% albumin= two studies	28-and 90-day mortality
Sivapalan et al. (2023) [21]	Meta-analysis	Total= 3978					Vasopressor days and vasopressor-free days, RRT usage, RRT free days, Acute kidney injury incidence, Adverse events

Discussion

Corl et al. conducted a trial for restrictive intravenous fluids in septic patients [11]. The mean difference of the fluid administered in the first 24 hours after randomization was 586 ml, a relative reduction of 47% between the groups. The primary outcome of the trial was the 30-day all-cause mortality which showed no significant difference between the restrictive group (21.8%) and the usual care group (22.2%). The trial also showed that in patients who were intubated, restrictive fluid administration resulted in fewer hours on ventilator support as compared to usual care recipients, suggesting that the restrictive fluid strategy may play a role in limiting lung injury. There were no significant observed differences in organ dysfunction and adverse events between the two groups. The REFACED sepsis trial achieved a 58% relative reduction between the restrictive fluid and usual care groups [12]. The 30-day mortality showed no statistically significant differences between the two groups (14.8% in the restrictive group vs. 16.1% in the usual care group). The most common reason for fluid administration in the restrictive group was hypotension (systolic blood pressure < 90mmHg). This trial concluded that a restrictive fluid strategy is a viable option in patients in septic patients without shock as the rates of 90-day mortality, and new-onset kidney failure were comparable between the groups. The CLOVERS trial focused on a group of patients with sepsis-induced hypotension [13]. The trial detected no statistically significant difference in 90-day mortality between the restrictive and liberal fluid groups. Although the rate of serious adverse effects was similar between the two groups, there were higher rates of fluid overload and pulmonary edema in the liberal fluids group further showing the benefit of a restrictive fluid approach. There was a mean difference of 2314ml in administered fluids between the groups, a relative reduction of 62.7%. A trial conducted by Meyhoff et al. included patients who were in septic shock and had similar findings [14]. The 90-day mortality showed no significant differences between the restrictive and standard fluid groups. Both groups had similar mortality rates without ventilators and after hospital discharge. The rate of serious adverse effects in the restrictive group was 29.4% with severe acute kidney injury being the most common adverse effect (23.1%). The same was true for the standard fluid group which had 30.8% serious adverse effects of which severe kidney injury was the most common (24.5%). These trials showed that the rate of survival and adverse events was comparable between the restrictive and liberal fluid groups, but the CLOVERS trial showed the added benefit of fluid restriction with lower rates of fluid overload-related complications.

Brown et al. [15] conducted a secondary analysis of the SMART trial [16]. The SMART trial included all critically ill patients admitted to the ICU but Brown et al. only included the data from the patients that were admitted with the diagnosis of sepsis. This study found that, compared to saline, balanced crystalloid use led to a lower rate of 30-day mortality (26.5% vs. 31.2%), greater vasopressor-free days (27 vs. 28), and fewer major adverse kidney events (35.4% vs. 40.1%). The study by Jackson et al. was another secondary analysis of the SMART trial [17]. This study had two categories, an ICU-only period where the choice of fluid was chosen in the ICU and the emergency department (ED) and ICU period in which the choice of fluid was decided at the time of arrival in the ED. The incidence of 30-day mortality between the balanced crystalloid and saline groups was comparable when the fluid choice was made in the ICU only but when the choice of fluid was made in the ED, the incidence of 30-day mortality between groups showed a greater difference (24.9% vs. 30.6%). The same was true for ventilator, vasopressor, and ICU-free days. The balanced crystalloids group had fewer new onsets of renal replacement therapy among the patients regardless of when the choice of fluid was made. This trial showed that the administration of balanced crystalloids early in sepsis has a greater effect on patient survival.

The ALBIOS study [18] was a trial to compare the safety and efficacy of albumin replacement in patients with sepsis [18]. The trial found that albumin replacement was safe and that it provided hemodynamic advantages. A greater number of patients in the albumin group reached the mean arterial pressure target within six hours of randomization. Albumin replacement also led to a lower incidence of 90-day mortality in a sub-group of patients with septic shock but provided no added benefits for the 28 and 90-day mortality for patients with severe sepsis. Zhou et al. conducted a retrospective observational study comparing the effects of early administration of a combination of albumin and crystalloids [19]. The study included 6597 patients in the crystalloid alone group and 920 patients in the combination group. The study found that administration of a combination of albumin and crystalloids provided a mortality benefit. The restricted mean survival time (RMST) at day 28 was 22.20 days for crystalloids and 25.10 days for combination, a difference of 3.32 days in favor of the combination group. RMST at day 60 was 42.18 days for crystalloids and 50.93 days for combination, with a mean difference of 9.09 days again in the favor of combination group. In addition, compared to the early (0-24 hours) combination, preceded (<0 hours) and late (>24 hours) showed a lesser increase in survival.

Geng et al. conducted a meta-analysis of randomized controlled trials (RCTs) comparing crystalloids and different concentrations of albumin [20]. There was no statistically significant difference in mortality at day 28 between treatment with albumin and crystalloids. For 90-day mortality, the treatment with albumin resulted in a lower rate compared to crystalloids for patients with septic shock but not for those with severe sepsis. This meta-analysis found, through probability-based ranking, that 4-5% albumin may lead to lower 28-day mortality compared to 20% albumin and crystalloid for patients with severe sepsis. In comparison, 20% albumin reduced the 28-day mortality in patients with septic shock showing that albumin is more effective than crystalloids in reducing the mortality rate.

Sivapalan et al. conducted a meta-analysis comparing liberal and restrictive fluid strategies for patients with sepsis [21]. This meta-analysis found no statistically significant differences in mortality, RRT use, acute kidney injury, and vasopressor-free days. Furthermore, the analysis found no difference in adverse events between the two strategies.

Limitation

This review has several limitations. Firstly, four trials included in this review were unblinded and multiple trials reported a breach of protocol which may have led to the introduction of bias. Secondly, fluid management guidelines differ between centers which could have influenced the safety and efficacy results. Thirdly, some included studies did not take into account the co-morbidities of the included population which could have influenced the results [22,23]. Fourthly, the included studies had an enrollment of the trial population with different severity of sepsis which could have affected the outcomes. Lastly, different endpoints were reported by different RCTs that affected the overall interpretation.

## Conclusions

In conclusion, this review shows that liberal fluid administration and restrictive fluid administration have comparative safety and efficacy results. As compared to normal saline, balanced crystalloids resulted in lower mortality and were associated with fewer incidences of kidney injury. Albumin replacement was found to be safe and more effective than crystalloids and led to a higher rate of survival especially in patients with septic shock. Due to the limitations of this study and the low quality of data available, future studies are needed to further understand the factors that influence the efficacy of various fluid resuscitative strategies for patients with sepsis and septic shock.
